# Antiviral Development for the Polio Endgame: Current Progress and Future Directions

**DOI:** 10.3390/pathogens13110969

**Published:** 2024-11-06

**Authors:** Hang Xie, Eric E. Rhoden, Hong-Mei Liu, Folake Ogunsemowo, Bernardo A. Mainou, Rachel M. Burke, Cara C. Burns

**Affiliations:** 1 Poliovirus & Picornavirus Branch, Division of Viral Diseases, National Center for Immunization and Other Respiratory Diseases, Centers for Disease Control and Prevention, Atlanta, GA 30329, USA; imb0@cdc.gov (E.E.R.); hdl1@cdc.gov (H.-M.L.); wcm0@cdc.gov (F.O.); qlk6@cdc.gov (B.A.M.); zqd1@cdc.gov (C.C.B.); 2 Bill & Melinda Gates Foundation, Seattle, WA 98109, USA; rachel.burke@gatesfoundation.org

**Keywords:** poliovirus, immunodeficiency-associated vaccine-derived poliovirus, picornavirus, antiviral development, small molecule-based antiviral drug, capsid-binding agent

## Abstract

As the world is approaching the eradication of wild poliovirus serotype 1, the last of the three wild types, the question of how to maintain a polio-free world becomes imminent. To mitigate the risk of sporadic vaccine-associated paralytic polio (VAPP) caused by oral polio vaccines (OPVs) that are routinely used in global immunization programs, the Polio Antivirals Initiative (PAI) was established in 2006. The primary goal of the PAI is to facilitate the discovery and development of antiviral drugs to stop the excretion of immunodeficiency-associated vaccine-derived poliovirus (iVDPV) in B cell-deficient individuals. This review summarizes the major progress that has been made in the development of safe and effective poliovirus antivirals and highlights the candidates that have shown promising results in vitro, in vivo, and in clinical trials.

## 1. Introduction

Poliomyelitis (polio) is a highly contagious and debilitating disease caused by poliovirus. Poliovirus has three serotypes; each can cause poliomyelitis in humans. Poliovirus infects human cells via the specific poliovirus receptor—CD155—and replicates primarily in the intestines [1]. It can also spread to the central nervous system, resulting in paralysis. The mode of transmission is primarily via the fecal–oral route and, less frequently, via contaminated food or water. Neonates, infants, and children under 5 years of age are the most vulnerable populations for polio [2]. In most cases, infected children have mild cold-like symptoms and can recover without medical intervention [3]. However, in rare cases (<1–2%), patients can suffer neurological damage and develop permanent paralysis or even die [3]. Since the late 1980s, the Global Polio Eradication Initiative (GPEI) has helped to distribute and administer over three billion doses of live attenuated oral polio vaccines (OPVs or Sabin vaccines) to children living in developing countries, achieving greater than a 99% reduction in global polio cases [2]. As both serotype 2 and serotype 3 wild polioviruses (WPVs) have been declared eradicated globally, and serotype 1 WPV remains in circulation in only two countries (Afghanistan and Pakistan) [4], we have never been so close to a polio-free world.

OPVs induce mucosal immunity against WPVs via limited replication in the gastrointestinal tract [5,6,7]. However, OPVs can also potentially cause vaccine-associated paralytic polio (VAPP), a rare adverse event when an administered OPV reverts to neurovirulence, resulting in paralysis in some recipients or unimmune persons in close contact [8,9,10]. The risk for VAPP has been estimated at 4.7 cases per million births globally, with incidents more frequently reported in recipients living in low-income countries who had taken more than three OPV doses and those from middle/high-income countries who were unvaccinated or who had taken only one OPV dose [11]. The persistent replication of an OPV in the gut could also lead to the reversion of virulence and allow the shedding of vaccine-derived polioviruses (VDPVs), which can spread among populations with low vaccine coverage, leading to circulating vaccine-derived polioviruses (cVDPVs) that can cause outbreaks of paralysis [12,13]. Moreover, B cell-deficient immunocompromised persons can become chronically infected, resulting in immunodeficiency-associated VDPV (iVDPV) [14,15,16,17]. Hence, these vaccine-related viruses pose great threats to a polio-free world, even after the eradication of WPVs.

In 2006, the Polio Antivirals Initiative (PAI) was established at the Task Force for Global Health as a multi-partner collaboration including the Bill & Melinda Gates Foundation, the World Health Organization (WHO), the US Centers for Disease Control and Prevention (CDC), the US National Institutes of Health (NIH), the US Food and Drug Administration (FDA), Rotary International, and the Jeffrey Modell Foundation. The PAI’s primary goal is to develop safe and effective antiviral drugs to stop vaccine poliovirus shedding in immunodeficient recipients of OPVs and to mitigate the threat of prolonged poliovirus excretion to a polio-free world after the endgame (https://www.taskforce.org/polio-antivirals-initiative/ (accessed on 6 October 2024)).

This review summarizes the major efforts made in the discovery of anti-poliovirus small molecules and the progress achieved in clinical development. It also discusses the challenges encountered along the way and suggests future directions that may facilitate the discovery and development of more effective antivirals for treating infections associated with cVDPV, iVDPV, or the accidental release of other live polioviruses during or after the endgame.

## 2. Poliovirus Biology

Poliovirus belongs to the genus Enterovirus in the Picornaviridae family of 158 species, including many viruses (e.g., coxsackieviruses, echoviruses, hepatitis A virus, and rhinoviruses) that are known to cause significant human diseases. All picornaviruses are nonenveloped and have an icosahedral protein coat of 30 nm in diameter encapsidating a small, single-stranded positive-sense RNA genome [18].

**Figure 1:**
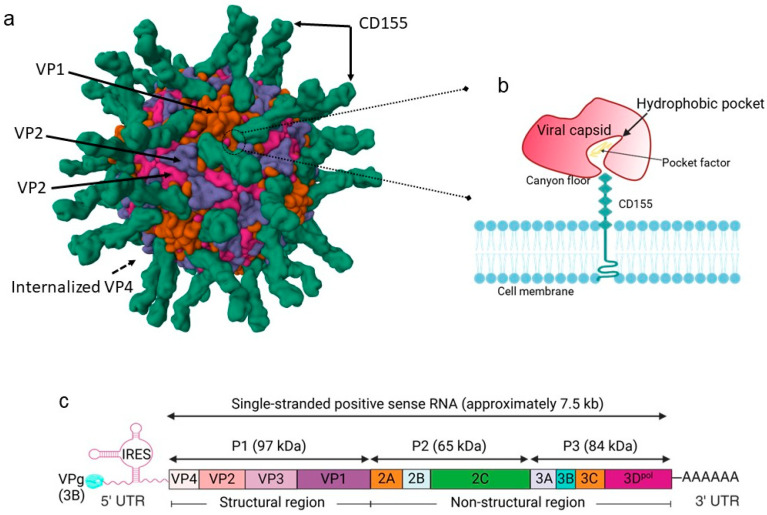
Poliovirus structure and viral genome and polyprotein organization. (**a**) Cryo-EM structure of poliovirus serotype 1 complexed with three domain CD155. Image from the RCSB PDB (RCSB.org) of PDB ID 1DGI [27]. (**b**) Schematic illustration of hydrophobic pocket in the viral capsid filled with pocket factor. (**c**) Organization of poliovirus genome and viral polyprotein processing (images in (**b**,**c**) were created with BioRender.com).

There are three serotypes of poliovirus that are differentiated by the antigenic sites in the viral capsid protein [19]. All three serotypes are very similar in the structure of the viral capsid protein. The poliovirus capsid has 60 protomers assembled in an icosahedral structure, each containing four polypeptides (VP1, VP2, VP3, and VP4) arranged in 12 identical pentamers [20] (Figure 1a). VP4 is located on the inner side of the capsid shell in contact with the viral genome, while VP1–VP3 form star-shaped mesas covering the external surface of the shell at the five-fold axes, surrounded by deep canyons and three-bladed propellers [21]. In poliovirus, a hydrophobic pocket is formed under the canyon floor surrounding each five-fold axis of the viral capsid, which is usually occupied by one or more fatty acid-like natural ligands (pocket factors) and is accessible through a small pore [22] (Figure 1b). The interaction of the pocket factor with the hydrophobic pocket impacts virion thermal stability, and a point mutation (Phe-to-Leu) at residue 1134 of the ligand-binding site alters the temperature sensitivity of the Sabin 3 poliovirus [23]. The receptor-mediated poliovirus attachment induces a series of structural rearrangements in viral particles for subsequent uncoating and viral RNA (vRNA) transport and alters viral particle sedimentation from 160 S (mature virion) to 135 S (A particle) [24,25,26]. Following the translocation from the interior of the mature virion, the N-terminus of VP1 and myristoyl-VP4 become externalized in the 135 S particle to allow liposome binding and the formation of ion channels in lipid bilayers [24,25]. The 130 S particles are the intermediates in the process of receptor-mediated virus entry and are sensitive to proteases. Subsequent proteolytical removal of the first 31 amino acids of VP1 blocks the membrane association of the 135 S particles and allows vRNA release via the translocation pore in the bilayer, resulting in empty viral particles that sediment at 80 S [24,25,26].

De novo vRNA synthesis and protein translation produce a single large polyprotein that is cleaved and processed sequentially by viral proteases (2A, 3C, and 3CD) to yield P1, P2, and P3 precursors [28,29,30] (Figure 1c). The P1 precursor is further cleaved by viral protease 3CD to produce VP0, VP1, and VP3, which assemble into an empty capsid. VP0 is a myristoylated immature protein that is autocatalytically processed into myristoyl-VP4 and VP2 for vRNA encapsidation [31]. The P2 precursor is cleaved to produce viral protease 2A and non-structural proteins 2B and 2C [32]. The P3 precursor is split into 3AB and 3CD, followed by a proteolytical process, resulting in membrane binding 3A, RNA priming 3B (VPg), protease 3C, and RNA-dependent polymerase 3D (3D^pol^), respectively [30,32]. In addition to membrane association, viral proteins 2B, 2C, and 3A are also involved in replication complex formation, while viral protease 3C assists in capsid formation [33,34,35]. RNA primer VPg is covalently attached to the 5′ end of positive strand vRNA and is uridylylated by 3D^pol^ to form VPgpUpU for the initiation of vRNA synthesis [30,33] (Figure 1c).

## 3. Small Molecule-Based Antiviral Agents

### 3.1. Capsid-Binding Agents

Small molecule-based antiviral drug development for poliovirus has been largely focused on inhibiting virus replication by targeting the viral capsid or nonstructural proteins. Capsid-binding agents are the most extensively investigated antiviral class. These small molecules fill the hydrophobic pocket of the viral capsid by replacing the natural pocket factor. The replacement of the natural pocket factor by a small molecule increases the stability of the mature virion and prevents the capsid from undergoing conformational changes, thereby inhibiting the 135 S particle formation and blocking the viral uncoating for vRNA release [36,37,38,39,40].

**Pleconaril (VP-63843 or Picovir)** is an antiviral agent administered orally or intranasally for treating picornavirus-induced respiratory infections. It has high affinity and specificity for the hydrophobic pocket of the capsid and shows broad-spectrum antiviral activity against many common serotypes of enterovirus and rhinovirus in vitro [41]. Its predecessor, WIN 51711, was the first capsid inhibitor shown to prevent poliovirus-induced paralysis and death in mice following oral administration [42]. Pleconaril was used to treat an immunodeficient infant who acquired the poliovirus infection following an OPV administration, and it successfully eliminated Sabin 2-derived poliovirus from the cerebral spinal fluid and serum [43]. Additionally, Pleconaril combined with gamma globulin was effective in clearing prolonged poliovirus excretion in an immunocompromised child who developed VAPP after an OPV [44]. In another VAPP case, however, pleconaril in combination with immunoglobulin failed to stop virus excretion. A sequencing of virus isolates revealed the acquisition of a mutation (^261^Met to ^261^leu in VP1) adjacent to the binding site of pleconaril, leading to pleconaril resistance [45]. While pleconaril was shown to significantly reduce the duration of symptoms from a rhinovirus infection in two large placebo-controlled trials [46], concerns about potential drug–drug interactions (e.g., with birth control pills) have halted the further development of pleconaril to treat common colds associated with the rhinovirus infection [46]. In a randomized, double-blind, placebo-controlled trial of pleconaril for the treatment of neonatal enterovirus sepsis, an intent-to-treat analysis showed that the pleconaril-treated group had a significantly higher cumulative survival probability over 2 months (23%) than the placebo group (44%, *p* = 0.02) [47]. Hence, pleconaril was administered under compassionate use for treating children with life-threatening enterovirus infections for a few years [48,49].

**Pirodavir (R77975)** is another well-characterized capsid-binding agent originally developed for treating rhinovirus-induced common colds [50]. Pirodavir inhibits the replication of both serotype 2 and 3 polioviruses in vitro, but it is five- to twenty-fold less effective against serotype 1 poliovirus [51]. The pirodavir analog (R78206) binds the viral capsid in a single predominant conformation with its benzoyl ester group exposed at the open end of the pocket and exhibits stronger inhibitory effects on all three poliovirus serotypes, with half maximal effective concentration (EC_50_) values ranging from 0.11 to 0.76 µmol/L [21,51]. However, pirodavir and its analogs are sensitive to hydrolysis of the ester bond and exhibit poor pharmacokinetics as orally administered drugs [52,53], which limits their potential as anti-poliovirus agents.

**Pocapavir (V-073 or SCH 48973)** and its derivatives were first developed for treating nonpolio enterovirus infections [39,53]. Many analogs in this series share common characteristics with other capsid inhibitors in that they bind in the hydrophobic pocket of the viral capsid, resulting in increased thermal stability and inhibition of the early viral replication by blocking the uncoating process [38,39]. Pocapavir exhibits antiviral activity against a spectrum of enteroviruses in vitro without direct viricidal effects [38,39]. SCH 47802, an early lead compound in this series, had a 50% inhibitory concentration (IC_50_) of 0.04 μg/mL against serotype 2 poliovirus by a plaque reduction assay and was the only analog in the early development phase showing in vivo antiviral effects in a murine model of poliovirus-induced encephalitis [38]. Mice were protected from serotype 2 poliovirus-induced mortality when SCH 47802 was administered orally at 24 h before infection and continuously at a daily dose of 60 mg/kg for 15–21 days after infection [38]. However, other SCH 47802 derivatives in the early development phase lacked in vivo protective effects because of poor oral bioavailability, despite showing similar in vitro antiviral potency to SCH 47802 [38].

Pocapavir, a close analog of SCH 47802 with different substituting groups in the second o-chloro-p-metoxyphenol fragment, was also developed at Schering–Plough. Pocapavir has high affinity for the viral capsid, with an affinity constant of 8.85 × 10^−8^ M for poliovirus serotype 2, and it is potent against both serotypes 1 and 2 at IC_50_ of 0.02 μg/mL and 0.08 μg/mL in vitro, respectively [39]. ViroDefense, Inc. acquired SCH 48973 and renamed it V-073 and, later, pocapavir [54]. Unlike SCH 47802, which did not yield dose-dependent protection against poliovirus in vivo, orally administered pocapavir exerted a dose-dependent therapeutic effect in the range of 3, 10, and 20 mg/kg/day in mice infected with poliovirus serotype 2 [39]. Compared to a placebo, viral loads in the brain were 1–2 logs lower in mice receiving pocapavir orally four times at 5 mg/kg within 6–24 h post infection [39]. These results demonstrated that pocapavir has higher in vivo efficacy than SCH 47802, which is probably attributable to its better bioavailability, as SCH 47802 and other derivatives in this series are highly hydrophobic and show plateaued absorption in a range of pharmacokinetic concentrations [38].

Pocapavir has broad antiviral activity against polioviruses and inhibits 45 strains of all 3 serotypes, including WPVs, Sabin vaccine strains, cVDPVs, and iVDPVs [54]. Pocapavir shows a higher antiviral specificity for poliovirus (EC_50_ ranging 0.003–0.126 µM with mean EC_50_ value of 0.029 µM) than other nonpolio picornaviruses, such as echoviruses (EC_50_ ranging 0.009–7.08 µM with mean EC_50_ value of 0.86 µM), enteroviruses (EC_50_ ranging 0.236–14 µM with mean EC_50_ value of 3.47 µM), or coxsackieviruses (EC_50_ ranging 0.007–14 µM with mean EC_50_ value of 3.79 µM) in an in vitro cytopathic effect (CPE)-based assay [54]. Tanner et al. reported that poliovirus titers were significantly decreased in the muscles of infected Tnfr1−/− PVR+/+ transgenic mice following daily pocapavir treatment at 10 mg/kg initiated after infection, and no drug-resistant variants were selected during the treatment [55].

Pocapavir has been used to treat several cases of iVDPV in pediatric patients under expanded access (ViroDefense unpublished data). In an infant iVDPV case with the presence of neurovirulence and prolonged excretion, pocapavir was administered by mixing it with the infant formula once per day at a dose of 27 mg/kg body weight for 14 consecutive days [56]. Poliovirus shedding in stools stopped as early as Day 2, and negativity was maintained through 7 weeks posttreatment, when the last sample was tested. The infant tolerated pocapavir well during the course of treatment, without any adverse events reported [56].

In a monovalent oral poliovirus serotype 1 vaccine (mOPV1) placebo-controlled human challenge trial involving 141 healthy adults who had received a childhood inactivated poliovirus vaccine (IPV), the median clearance time was significantly shortened in pocapavir-treated subjects (10 days) compared to placebos (13 days) (*p* = 0.0019) [57]. However, a resistant virus was detected in the stools of 44% (41 out of 93) of the pocapavir recipients as well as 10% (5 out of 48) of the placebos [57]. The generation of pocapavir-resistant polioviruses in placebo-treated subjects indicates that there was significant virus transmission among subjects in the study unit.

In in vitro drug-resistance studies, pocapavir-resistant poliovirus variants emerged at the approximate frequency of 3–40 × 10^−5^, with the majority of viruses bearing a point mutation in either VP1 with ^194^Ile to ^194^Met or ^194^Phe (equivalent position 192 in serotype 3) or VP3 with ^24^Ala to ^24^Val (Table 1 and Table 2) [58]. Both residues are within the hydrophobic pocket that is targeted by pocapavir. BTA798, a capsid inhibitor used for treating human rhinovirus infections, was able to inhibit the replication of pocapavir-resistant strains bearing the mutation of ^24^Ala to ^24^Val in VP3, but the combination of two capsid inhibitors only yielded the additive inhibition on poliovirus [59]. Although pocapavir is still available for compassionate use, the frequency of drug-resistance development in vitro and in the human challenge study suggests that pocapavir may be best used in combination with other antivirals with different mechanisms of action to treat immunodeficient poliovirus excreters.

**H1PVAT** is another capsid-binding agent that selectively inhibits all three poliovirus serotypes but does not affect other enteroviruses [60]. Drug-resistance results have revealed that all H1PVAT-resistant variants uniformly bear a single ^194^Ile to ^194^Phe mutation in VP1 [60], suggesting that H1PVAT may have a similar mechanism of action as pocapavir. Additional investigations are needed to determine whether H1PVAT has advantages over other small-molecule inhibitors to justify further advancement.

### 3.2. Protease 3C Inhibitors

**Rupintrivir (AG7088)** is an irreversible pan-3C protease inhibitor designed by Pfizer using a protein structure-based drug design strategy [61]. Rupintrivir contains peptidyl-binding elements specific for the 3C protease and an unsaturated ethyl ester Michael acceptor that can interact with the cysteine residue in the active site of the 3C protease to form an irreversible covalent bond resulting in the inactivation of the 3C protease [61]. Rupintrivir shows broad in vitro antiviral activities against human rhinoviruses, coxsackieviruses, enteroviruses, and echoviruses [62]. Rupintrivir also potently inhibits the replication of all three poliovirus serotypes, with EC_50_ values ranging between 5 and 40 nM [51]. Despite the high in vitro potency, the failure of rupintrivir to reduce the severity of natural rhinovirus infections in humans has halted its clinical development [63,64].

**Imocitrelvir (V-7404)** is also an irreversible protease 3C inhibitor originally developed by Pfizer for treating human rhinovirus infections [65]. This molecule, currently under development by ViroDefense, Inc., shows improved pharmacokinetic properties compared to its predecessor rupintrivir (known as Compound **1** in early studies) and has demonstrated good oral bioavailability in dogs, cynomolgus monkeys, and humans [63,66]. Compared to rupintrivir, imocitrelvir is less potent against poliovirus of all three serotypes, with EC_50_ values ranging between 0.080 and 0.674 μM [59]. However, imocitrelvir is active against all pocapavir-resistant variants, with EC_50_ values (0.218 to 0.819 μM) comparable to those (0.202–0.407 μM) for pocapavir-susceptible parent strains [59]. The available data indicate that the emergence of laboratory-derived imocitrelvir-resistant variants was at the approximate frequency of 4–8 × 10^−5^ (Table 1 and Table 2), similar to that (3–40 × 10^−5^) of laboratory-derived pocapavir-resistant variants in vitro [58]. Importantly, despite bearing a single ^165^Gly to ^165^Ser mutation in the 3C protease, imocitrelvir-resistant VDPV1 variants remain susceptible to pocapavir (EC_50_ value of 0.044–0.046 μM) comparable to that of the parent VDPV1 strains (EC_50_ value of 0.038 μM) (Table 1).

### 3.3. Imocitrelvir/Pocapavir Combination Therapy

Given that imocitrelvir-resistant variants are sensitive to pocapavir, our team tested the combination of the two drugs against poliovirus in vitro. The combination of imocitrelvir and pocapavir blocked CPE formation induced by Sabin 1, 2, and 3 [59]. Strong in vitro synergy was achieved when the combination was used to treat parental VDPV1 and imocitrelvir-resistant VDPV1 variants. Although weak synergistic effects were observed when imocitrelvir and pocapavir were used to treat pocapavir-resistant VDPV1 variants, imocitrelvir remained active against these pocapavir-resistant variants. As expected, no synergistic activity was observed on pocapavir/imocitrelvir double-resistant VDPV1 variants treated with the combination (Table 1). Pocapavir/imocitrelvir double-resistant VDPV1 variants emerged at a mean frequency of 1.9 × 10^−7^ in vitro, which is >100 times lower than that observed with VDPV1 variants resistant to either drug alone (Table 1). Laboratory-isolated double-resistant VDPV1 variants have comparable growth kinetics to their drug-sensitive parental strains in vitro but are more thermolabile (CDC unpublished data). The emergence of pocapavir/imocitrelvir double-resistant VDPV2 or VDPV3 variants is even less frequently detected in cell culture (<1 × 10^−9^, the detection limit of the assay) (Table 2). The potent synergy and markedly reduced resistance frequency, taken together, suggest that imocitrelvir paired with pocapavir as a combination therapy could be effective for treating infections with cVDPVs or iVDPVs.

ViroDefense, Inc. has completed a Phase I double-blind, randomized, placebo-controlled clinical trial on the safety, tolerability, and pharmacokinetics of imocitrelvir alone and in combination with pocapavir orally administered in healthy adult volunteers. Imocitrelvir and its combination with pocapavir were found to be well tolerated by the subjects, with acceptable safety and pharmacokinetics profiles, after a single ascending dose or multiple ascending doses [67]. This study also provided valuable dosing data for imocitrelvir to be paired with pocapavir for the further clinical development of the combination therapy.

### 3.4. Protein 2C Inhibitors

Poliovirus 2C is one of the highly conserved nonstructural proteins. It has ATPase/GTPase activity and plays an important role in RNA binding, membrane binding, and viral morphology [35,68,69,70]. Guanidine hydrochloride (GuaHCl), a reversible 2C inhibitor, can impair poliovirus replication by blocking 2C and its precursor P2 to become membrane-bound, abolishing 2C ATPase activity and preventing the initiation of negative-stranded RNA synthesis [68,69,70]. Other 2C inhibitors such as 2-(a-hydroxybenzyl)-benzimidazole (HBB) and its derivative, 1-(4-fluorophenyl)-2-[(4-imino-1,4-dihydropyridin-1-yl)methyl] (MRL-1237), also reportedly inhibit poliovirus replication but are prone to drug resistance due to spontaneous mutations [71]. Most laboratory-derived MRL-1237-resistant variants are also resistant to GuaHCl [71], suggesting that these 2C inhibitors share a common mode of action. The high tendency toward drug resistance excludes 2C inhibitors from the list of potential lead antiviral candidates for further development.

### 3.5. Protein 3A Inhibitors

Poliovirus 3A is another highly conserved, multifunctional nonstructural protein involved in virus–host interactions, such as membrane association, host secretory pathways, RNA replication organelles, etc. [64,72,73]. Poliovirus 3A and its precursor, 3AB, can suppress host phosphatidylinositol-4 kinase III beta (PI4Kβ) and oxysterol-binding protein (OSBP) pathways to modulate the lipid microenvironment for the generation of viral RNA replication organelles [74,75,76].

Enviroxime, a benzimidazole derivative originally developed to treat rhinovirus-induced symptoms, inhibits the replication of all three poliovirus serotypes by targeting the 3A coding region [77]. However, single point mutations in this 3A coding region are sufficient to abolish the inhibition of enviroxime and other enviroxime-like compounds (e.g., TTP-8307, T-00127-HEV1, and GW5074) [64,77,78,79]. Additionally, enviroxime combined with BTA798 (capsid inhibitor) or rupintrivir (3C protease inhibitor) yielded only an additive or very weak synergistic inhibition of poliovirus [80]. In clinical studies, undesirable gastrointestinal side effects were reported in some subjects receiving an oral administration of enviroxime, and no apparent efficacy was observed in natural or experimental rhinovirus infections [64,77,81]. Because of poor pre-clinical data, the clinical development of enviroxime has been halted.

### 3.6. Polymerase 3D^pol^ Inhibitors

Poliovirus polymerase 3D^pol^ plays a key role in catalyzing viral RNA synthesis, which can be blocked by non-nucleoside and nucleoside analogs. Guanidine is a historic non-nucleoside inhibitor that blocks the RNA synthesis of all three poliovirus serotypes [82]. Gliotoxin, a fungal metabolite, is another non-nucleoside inhibitor that interferes with poliovirus RNA synthesis by targeting 3D^pol^ [83]. However, both guanidine and gliotoxin have toxicities that prevent them from further clinical development.

Ribavirin, a nucleoside inhibitor with a multimode mechanism of action, is a regulatory-approved drug for treating RSV and chronic HCV infections [84]. As a synthetic purine nucleoside analogue, ribavirin can act as an ambiguous purine base inhibitor that is incorporated into the poliovirus RNA genome, resulting in lethal mutagenesis and blocking the generation of progeny virus [85,86]. As ribavirin increases the number of mutations per genome, the infectivity of the resulting poliovirus RNA drops sharply [86]. However, the spontaneous RNA recombination that drives the natural evolution of the picornavirus is also suggested to contribute to ribavirin resistance [87]. Poliovirus containing ^420^Leu in 3D^pol^ has higher frequencies of RNA recombination and is more resistant to ribavirin than poliovirus bearing ^420^Ala mutation in 3D^pol^ [87].

### 3.7. New Antiviral Leads

In addition to the above antiviral drugs and candidates that have been tested or developed in the past decades, there are compounds that have recently been discovered to have antiviral activities against poliovirus. Madia et al. reported that two new oxazoline derivatives are highly potent against poliovirus of all three serotypes at submicromolar concentrations, with very low cytotoxicity [88]. These isoflavone-derived synthetic compounds likely belong to the class of capsid inhibitors by impairing virus-receptor binding or viral uncoating, but the exact mechanism(s) remain to be elucidated [88].

A novel druggable interprotomer pocket in the capsid has been discovered that is located at a conserved VP1–VP3 interprotomer interface and is conserved across multiple species of picornaviruses [89]. Preliminary structure–activity relation studies have identified several analogs targeting this pocket with broad-spectrum antiviral activities against different picornaviruses, including one against poliovirus serotype 1, with low potency [89]. Further refining of the functional groups of these early-stage inhibitors may help improve antiviral activity.

Itraconazole, an antifungal agent with anticancer activity, also has antiviral effects on enteroviruses, including poliovirus [90]. Itraconazole targets host OSBP and OSBP-related protein 4 (ORP4) and impairs the colocalization of host filipin with viral 3A to block viral replication organelle formation [90]. Although itraconazole itself does not directly inhibit PI4KIIIβ activity, mutations in the 3A protein that confer resistance to enviroxime, GW5074, or other PI4KIIIβ inhibitors also render viruses resistant to itraconazole [90].

## 4. Summary and Future Directions

Although extensive progress has been made in developing antiviral drugs targeting poliovirus in the past decades, none has achieved regulatory approval for therapeutic use. Poor pharmacokinetics, low oral bioavailability, toxicity, and drug resistance stand among the many issues that hinder clinical development. The standards for safety, tolerability, and oral bioavailability could be even higher for polio antiviral drug approval, considering that the potential target population is primarily children under 5 years old and immunocompromised individuals. Another significant factor is the as-yet-undefined regulatory pathway to approval for a rare condition such as iVDPV excreters.

Drug development from de novo molecule design to clinical investigation to final approval is a long, costly journey full of uncertainties even when the pathway is reasonably well defined. The entire process can easily span 10–15 years or more, from conceiving an idea to achieving the final approval. The approach of drug repurposing, which is to reinvestigate existing drugs for new applications, has gained considerable interest during the COVID-19 pandemic. In addition to reducing time and saving money, the largest benefit of repurposing licensed drugs is the ability to take an accelerated approval path for new indication(s) because licensed drugs have already passed the toxicity tests, and many have post-licensure or post-marketing surveillance data available. This approach can significantly shorten the antiviral development cycle. One such successful example is azidothymidine (AZT). Originally developed for anti-cancer treatment, AZT was discovered by the US NIH/NCI-led drug screening program to be a potent antiretroviral agent and was approved by the US FDA as a therapeutic drug for treating HIV infections in 1987 after fast-track clinical trials [91]. Remdesivir is a drug approved for treating patients with COVID-19-associated hospitalizations. Remdesivir’s intracellular metabolites GS-441524 monophosphate and triphosphate are the nucleotide analogs of the viral RNA polymerase inhibitor [92]. Our team tested the potential of remdesivir and its metabolites in inhibiting poliovirus replication without success (CDC unpublished data).

In addition to repurposing licensed drugs, the pools of screened molecules can be expanded to include compounds under investigation or in databases. Previously, this strategy helped to successfully identify a synthetic agonist of peroxisome proliferator-activated receptor β/δ as a promising lead compound with broad-spectrum anti-influenza activity in vitro and in ovo after screening over 70,000 compounds in the LOPAC and Maybridge libraries [93]. Although candidate drugs selected by this approach may still need to proceed through the regular approval process, it will save considerable time in compound design and synthesis. Additionally, artificial intelligence, such as machine learning-driven frameworks, should be leveraged for more efficient drug selection and optimization. Single cell-led systems biology pipelines and other emerging technologies could also be applied to facilitate the identification of promising candidates [94].

In summary, despite the challenges and obstacles, there is progress in the development of antiviral agents that are essential in stopping the excretion of iVDPV in immune-deficient patients. At present, the most promising prospect is the combination of pocapavir and imocitrelvir (which shows synergistic antiviral activity and a very low resistance rate in vitro), which has progressed through the early stages of non-clinical and clinical development.

## Figures and Tables

**Table 1:** Susceptibility of selected poliovirus serotype 1 strains to pocapavir, imocitrelvir, or combination treatment *.

Virus	Serotype	Mutation(s)	Pocapavir		Imocitrelvir		Imocitrelvir/Pocapavir		Reference(s)
			EC_50_ (µM)	Resistance Frequency	EC_50_ (µM)	Resistance Frequency	Synergy (%) ^#^	Resistance Frequency	
Sabin 1	Serotype 1, Vaccine strain	-	0.017–0.025	1.9 × 10^−7^	0.173–0.541	Not determined	Strong (580 µM^2^)	Not determined	This review & Rhoden et al. (2013) [59]
10235-DOR-WT	cVDPV1	-	0.018–0.038	3.31 × 10^−5^	0.202–0.501	8.2 × 10^−5^	Strong (892 µM^2^)	1.9 × 10^−7^	This review & Liu et al. (2012) [58]
10235-DOR-1D	cVDPV1, Pocapavir-variant	^194^Ile to ^194^Phe in VP1	>10	Not determined	0.316–0.612	Not determined	Weak (47 µM^2^)	Not determined	This review
10235-DOR-3D	cVDPV1, Pocapavir-variant	^24^Ala to ^24^Val in VP3	>10	Not determined	0.287–0.657	Not determined	Weak (41 µM^2^)	Not determined	
10235-DOR-4G	cVDPV1, Imocitrelvir-variant	^165^Gly to ^165^Ser in 3C	0.034–0.058	Not determined	8.963–9.497	Not determined	Strong (820 µM^2^)	Not determined	This review
10235-DOR-6G	cVDPV1, Imocitrelvir-variant		0.027–0.061	Not determined	8.666–9.734	Not determined	Strong (834 µM^2^)	Not determined	
10235-DOR-3J	cVDPV1, Imocitrelvir/Pocapavir-variant	^24^Ala to ^24^Val in VP3 & ^165^Gly to ^165^Ser in 3C	>10	Not determined	>10	Not determined	Not determined	Not determined	This review
10235-DOR-4J	cVDPV1, Imocitrelvir/Pocapavair-variant		>10	Not determined	8.873–9.107	Not determined	No (1 µM^2^)	Not determined	
10235-DOR-9J	cVDPV1, Imocitrelvir/Pocapavir-variant		>10	Not determined	>10	Not determined	Not determined	Not determined	
10235-DOR-1I	cVDPV1, Imocitrelvir/Pocapavir-variant		>10	Not determined	>10	Not determined	Not determined	Not determined	

* Susceptibility of parental and drug-resistant variants was assessed by co-incubating virus with drug in a cross-titration format in HeLa or RD cells seeded onto 96-well plates. After 3 days of incubation at 37 °C, viral cytopathic effect was determined by crystal violet staining and measured by reading absorbance at 590 nm. cVDPV: circulating vaccine-derived polioviruses; EC_50_: half maximal effective concentration. ^#^ Synergistic effects of imocitrelvir and pocapavir were determined by cross-titration based on a checkerboard dilution matrix and calculated using MacSynergy II. The synergy volume expressed in µM^2^% is categorized as follows: strong (>100), moderate (>50–100), weak (25–50), and no (<25).

**Table 2:** Susceptibility of selected poliovirus serotype 2 and serotype 3 strains to pocapavir, imocitrelvir, or combination treatment *.

Virus	Serotype	Mutation(s)	Pocapavir		Imocitrelvir		Imocitrelvir/Pocapavir		Reference(s)
			EC_50_ (µM)	Resistance Frequency	EC_50_ (µM)	Resistance Frequency	Synergy (%) ^#^	Resistance Frequency	
Sabin 2	Serotype 2, Vaccine strain	-	0.016–0.024	<1 × 10^−9^	0.305–0.340	Not determined	Strong (459 µM^2^)	Not determined	This review & Rhoden et al. (2013) [59]
10230	cVDPV2	-	0.036–0.055	11.8 × 10^−5^	0.314–0.502	5.4 × 10^−5^	Not determined	<1 ×10^−9^	Liu et al. (2012) [58] & Rhoden et al. (2013) [59]
10230.4	cVDPV2, Pocapavir-variant	^194^Ile to ^194^Met in VP1	1.5	Not determined	0.292–0.344	Not determined	Not determined	Not determined	Liu et al. (2012) [58] & Rhoden et al. (2013) [59]
10230.8	cVDPV2, Pocapavir-variant	^24^Ala to ^24^Val in VP3	>10	Not determined	0.243–0.295	Not determined	Not determined	Not determined	Liu et al. (2012) [58] & Rhoden et al. (2013) [59]
Sabin 3	Serotype 3, Vaccine strain	-	0.020–0.048	<1 × 10^−9^	0.137–0.262	Not determined	Strong (288 µM^2^)	Not determined	This review & Rhoden et al. (2013) [59]
10805	iVDPV3	-	0.024–0.034	30.8 × 10^−5^	0.313–0.401	Not determined	Not determined	<1 × 10^−9^	Liu et al. (2012) [58] & Rhoden et al. (2013) [59]
10805.1	iVDPV3, Pocapavir-variant	^194^Ile to ^194^Phe in VP1	>10	Not determined	0.686–0.952	Not determined	Not determined	Not determined	Liu et al. (2012) [58] & Rhoden et al. (2013) [59]
10805.5	iVDPV3, Pocapavir-variant	^24^Ala to ^24^Val in VP3	1.1	Not determined	0.548–0.638	Not determined	Not determined	Not determined	Liu et al. (2012) [58] & Rhoden et al. (2013) [59]

* Susceptibility of parental and drug-resistant variants was assessed by co-incubating virus with drug in a cross-titration format in HeLa or RD cells seeded onto 96-well plates. After 3 days of incubation at 37 °C, viral cytopathic effect was determined by crystal violet staining and measured by reading absorbance at 590 nm. cVDPV: circulating vaccine-derived polioviruses; EC_50_: half maximal effective concentration. ^#^ Synergistic effects of imocitrelvir and pocapavir were determined by cross-titration based on a checkerboard dilution matrix and calculated using MacSynergy II. The synergy volume expressed in µM^2^% is categorized as follows: strong (>100), moderate (>50–100), weak (25–50), and no (<25).
